# Supplementary material for: Single-Walled vs. Multi-Walled Carbon Nanotubes: Influence of Physico-Chemical Properties on Toxicogenomics Responses in Mouse Lungs
Source: Nanomaterials (Basel). 2023 Mar 15;13(6):1059. doi: 10.3390/nano13061059 (PMC10057402; doi:10.3390/nano13061059)
Supplement: Supplementary file 1 [file nanomaterials-13-01059-s001.zip › nanomaterials-2267591-supplementary.pdf]

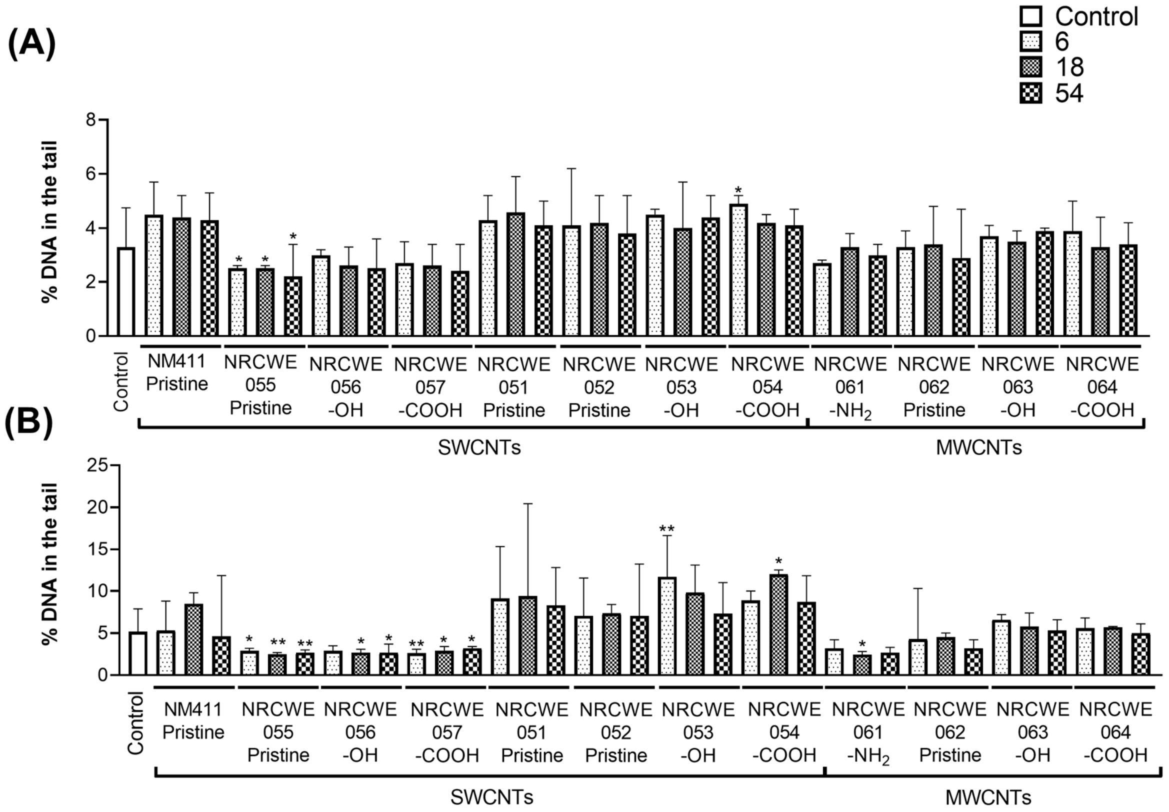

Figure S1. Percentage of DNA in the tail in lungs after exposure to individual SWCNTs or MWCNTs. (**A**) Data represent media and standard deviation and (**B**) median with interquartile range. Statistically significant differences between animals exposed and controls were determined through ANOVA (Dunnett's post-hoc) or Kruskal-Wallis (Dunn's post-hoc). *p < 0.05, **p < 0.01. (**A**) Day 1, (**B**) Day 28. Different colors and fill patterns indicate different dose groups: control, 6 μg/mouse, 18 μg/mouse and 54 μg/mouse of CNTs.

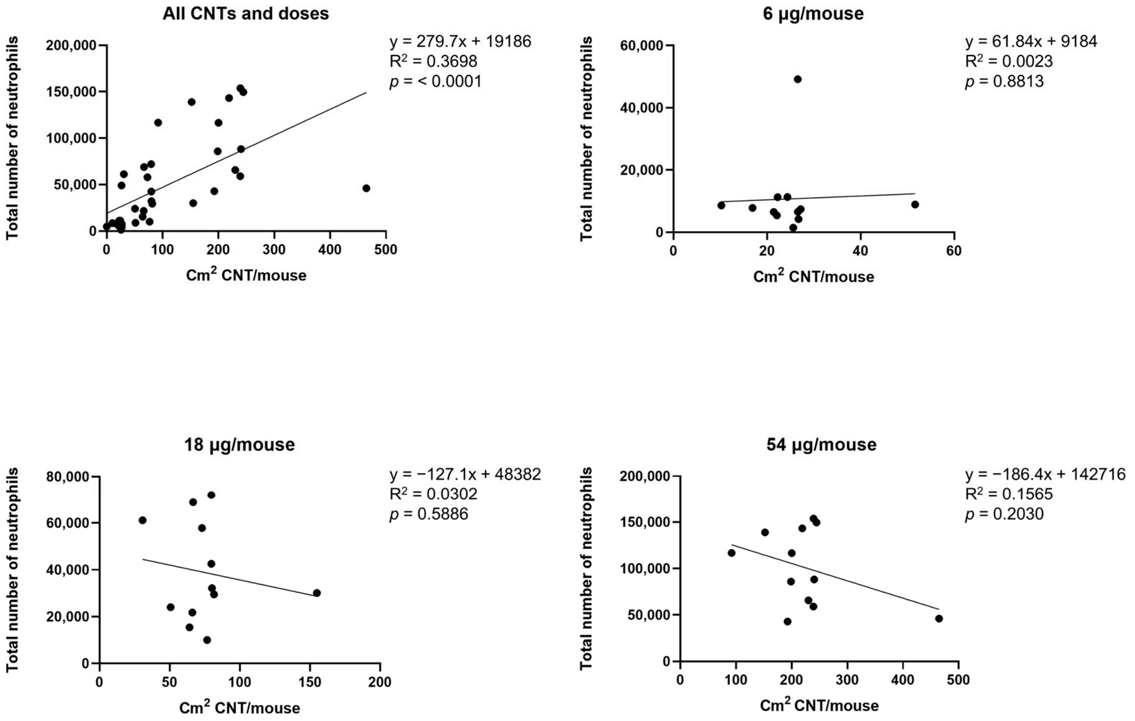

Figure S2. Relationship between the total number of neutrophils in the BALF and the total deposited surface area (BETxDose) on day 1 post-exposure to CNTs. Individual data points are the average total neutrophil numbers for each exposure group. Linear relationship was performed with GraphPad Prism 9.2.0.

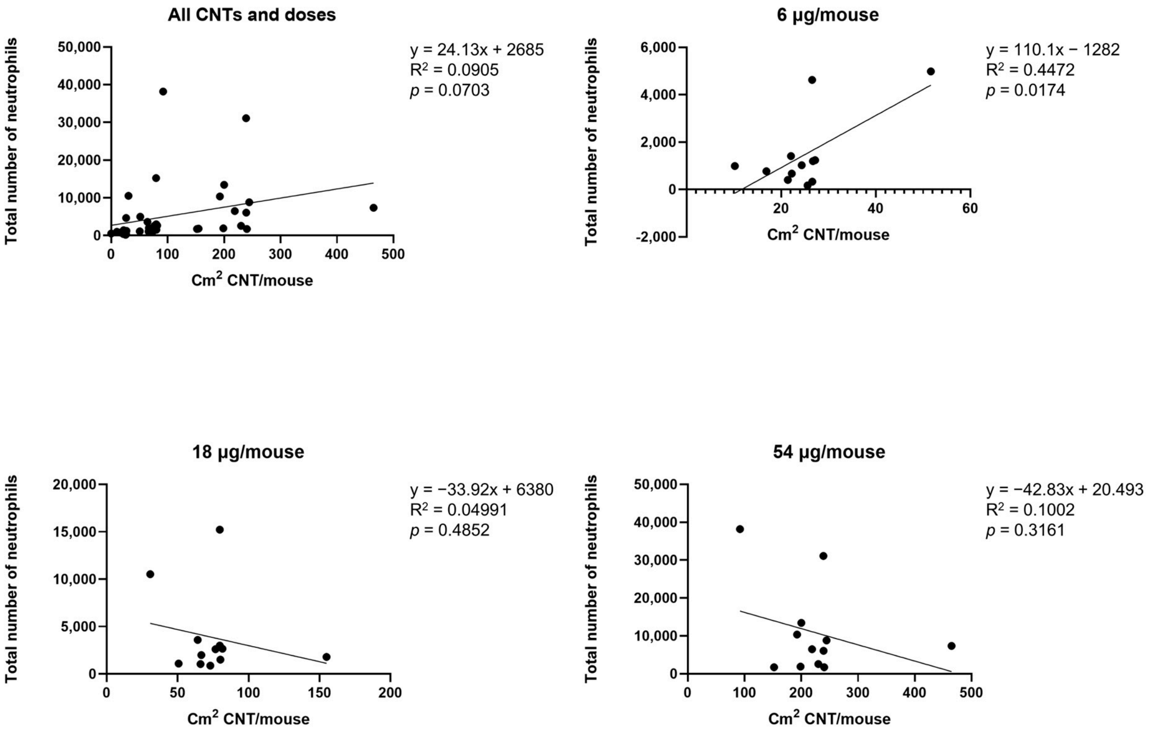

Figure S3. Relationship between the total number of neutrophils in the BALF and the total deposited surface area (BETxDose) on day 28 post-exposure to CNTs. Individual data points are the average total neutrophil numbers for each exposure group. Linear relationship was performed with GraphPad Prism 9.2.0.

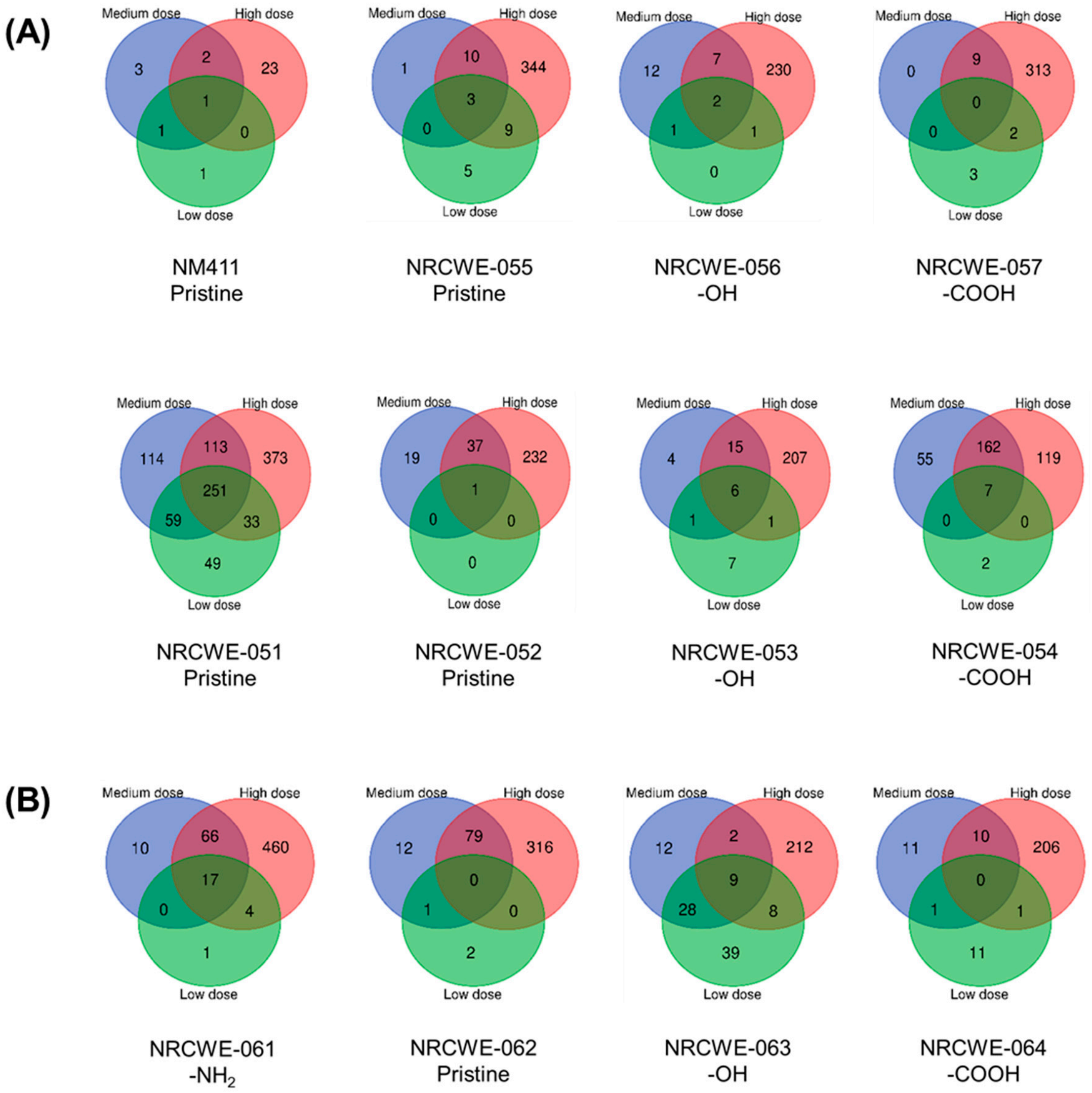

Figure S4. Venn diagrams showing the overlapping DEGs. (**A**) SWCNTs, (**B**) MWCNTs. DEGs were defined as genes with an FDR *p*-value ≤ 0.05 and fold change ±1.5 in either direction. Day 1 post-exposure. Low dose: 6 µg/mouse. Medium dose: 18 µg/mouse. High dose: 54 µg/mouse.

Overview of individual DEGs in each CNT group

DEGs for SWCNTs

NM-411 (SWCNTs-pristine): two DEGs were upregulated in a dose-dependent manner; *Ctk* showed 1.8 and 2-fold change; meanwhile, *Slc26a4* showed 5.5 and 9.7-fold change at the medium and high dose, respectively. At the highest dose, significant differential expression was observed for *Slc26a4* (9.7-fold), *Cxcl6* (8.9-fold), *C15orf48* (6.2-fold), *Ccl17* (5.4-fold), *Upk1a* (-1.7-fold), and *Tcf21* (-1.52-fold). *Ctsk* was the only DEG observed at the three doses (**Table S4a**).

NRCWE-055 (SWCNTs-pristine): three DEGs were upregulated at the three doses, *Bhlhe40* (1.5, 1.5, 1.5-fold), *Ctsk* (1.8, 2, and 2.2-fold), and *Gpnmb* (1.7, 2.1, 2.4-fold). In addition, at the low dose, *Chia* (2.1-fold), *Dbp* (2.5-fold), and *Srrm4os* (1.5-fold) were also upregulated, and *Fam220a* (-1.5-fold) and *Rasl11a* (-1.7-fold) were downregulated. Some DEGs were observed at the medium dose, and their fold change increased at the high dose, such as *Lcn2* (2.6, 4.1-fold), *Ly6a* (3.1, 5.7-fold), *Noxo1* (2.5, 4.2-fold), and *Sl26a4* (8.8, 12.5-fold). At the highest dose, significant fold changes were also observed for *Cxcl6* (17.1-fold), *Serpinb1c* (14.8-fold), *Saa1* (13.3-fold), *C15orf48* (8.4-fold), *Cc17* (8.2-fold), *Galnt15* (-2.3-fold), *Myh7* (-2.5-fold), *Fmo3* (-2.6-fold), *Bcan* (-2.6-fold), and *Fabp1* (-3.8-fold) (**Table S4b**).

NRCWE-056 (SWCNTs-OH): *Gpnmb* (1.8, 1.8, 2.4-fold) and *Serpinb1c* (6.7, 6.2, 14.3-fold) were upregulated at the three doses in a dose-dependent manner. In addition to those DEGs, three more DEGs were found at the low dose, *Cxcr1* (1.8-fold), *Gpnmb* (1.8-fold), and *5430402P08Rik* (1.6-fold). For medium dose, significant fold changes were observed for *Lcn* (2.3-fold), *Noxo1* (2.0-fold), *Ctsk* (1.9-fold), *Lypd6b* (1.7-fold), *Ripply3* (-1.7-fold), *Sox11* (-1.6-fold), *Sox4* (-1.6-fold),

and *Itga1* (-1.6-fold). At the highest dose, *Slc26a4* (9.8-fold), *Saa1* (6.7-fold), *Cxcl6* (6.5-fold), *Ccl7* (6.2-fold), *Cyp26b1* (-4.7-fold), *Fabp1* (-3.3-fold), *Slc4a1* (-2.4-fold), *Adrb3* (-2.2-fold), and *Amy2a* (-2.1-fold) were found with large fold changes values (**Table S4c**).

NRCWE-057 (SWCNTs-COOH): at the low dose, *Dbp* (2.2-fold), *Tex11* (1.6-fold), *Wee1* (1.5-fold) *Rasl11a* (-1.6-fold), and *Spon2* (-1.9-fold) exhibited differential expression. Some DEGs were upregulated in a dose-dependent manner, such as *Cxcl6* (8.2, 22.4-fold), *Slc26a4* (5.8, 17.3-fold), *Lcn2* (2.3, 4.1-fold), and *Ly6a* (2.2, 5.9-fold) at the medium and high dose. At the highest dose, large fold changes were observed for *Serpinb1c* (13.5-fold), *Reg3g* (12.3-fold), *Saa1* (10.3-fold), *C15orf48* (8.2-fold), *Ccl7* (7.7-fold), *Fabp1* (-2.6-fold), *Gsta5* (-2.5-fold), *Pon1* (2.1-fold), *Adrb3* (2.1-fold), and *Myh7* (-2.1-fold) (**Table S4d**).

NRCWE-051 (SWCNTs-pristine): Large fold changes were observed for some DEGs at the three doses, such as *Serpinb1c* (27.3, 37.9, 25.5-fold), *Cxcl6* (19.6, 23.2, 17.8-fold), *Slc26a4* (18.4, 18.2, 19.9-fold), *Cc17* (11.7, 13.6, 18.7-fold), *Fabp1* (-6.9, -7.1, -7.0-fold), *Dbp* (-2.5, -2.9, -3.6-fold), and *Myh7* (-2.8, -2.9, -3.2-fold). Moreover, large fold changes at the low dose were observed for *Cclca1* (25.3-fold), *Cyp26b1* (-4.0-fold), and *Asgr1* (-2.4). For medium dose, *Saa1* (16.4-fold), *Nr1d1* (-3.0-fold), *Fmo3* (-2.9-fold) were also found; meanwhile, at the highest dose, *Aldh1a3* (14.6-fold), *Gsta5* (-4.8-fold), and *Fmo3* (-6.0) were observed (**Table S5a**).

NRCWE-052 (SWCNTs-pristine): among the largest fold changes, two DEGs were upregulated in a dose-dependent manner at the medium and high dose, Serpinb1c (10.8, 27.5-fold) and *Slc26a4* (8.4, 14.8-fold). Some DEGs were found at medium dose, and their fold changes increased at the highest dose, such as C3 (1.9, 2.7-fold), Cc17 (2.8, 7.8-fold), *Cxcl6* (4.2, 5.3-fold), *Exo1* (1.5, 2.1-

fold), and *F10* (1.8, 2.4-fold). At the medium dose, *Cxcl6* (4.2-fold), *Ccl17* (4-fold), *Ly6a* (3.4-fold), *Sox7* (-1.6-fold), *Rgs2* (-1.6-fold), *Klra7* (-1.6-fold), *Sox4* (-1.6-fold) and *Fcrla* (-1.6-fold) were also found. Moreover, at the highest dose, *Saa1* (9.3-fold), *Orm1* (7.1-fold), *C15orf48* (6.2-fold), *Cyp26b1* (-5.3-fold), *Fabp1* (-3.7-fold), *Fmo3* (-2.8-fold), *Gsta5* (-2.8-fold), *Lrat* (-2.7-fold) were also observed to be differentially expressed (**Table S5b**).

<u>NRCWE-053 (SWCNTs-OH)</u>: some DEGs exhibited a response at the three doses tested, such as *Slc26a4* (4.5, 8.7, 19.4-fold), *Ccl17* (2.5, 3.9, 6-fold), *Cebpd* (2, 1.7, 1.6-fold), *Retnla* (1.8, 2, 2.3-fold), *2410022M11Rik* (-1.5, -1.6, -1.8-fold), and *Rgs2* (-1.5, -1.7, -1.6-fold). At the medium and high doses, the most upregulated and downregulated genes included *Serpinb1c* (10, 23-fold), *Cxcr1* (2.6, 2-fold), *Serpind1* (2, 1.7-fold), *Ctsk* (1.9, 2.4-fold), *Gpnmb* (1.9, 1.9-fold), *Dbp* (-2.7, -3.3-fold), *Nr1d1* (-2.0, -2.3-fold), and *Tef* (-1.5, -1.8-fold). At the lowest dose, significant fold changes were also observed for *Cstad* (-1.6-fold) and *Upk1a* (-1.5-fold); meanwhile, for the medium dose, *Dbp1* (-2.7-fold), *Nr1d1* (-2.0-fold), and *Tef* (-1.5-fold) were found downregulated. Large fold changes were also observed for *Cxcl6* (14-fold), *Saa1* (7-fold), *C15orf48* (6-fold), *Fmo3* (-4.6-fold), *Fabp1* (-3.6-fold), *Dbp* (-3.3-fold), *Gsta5* (-2.6-fold), *Pon1* (-2.5-fold) at the high dose (**Table S5c**).

<u>NRCWE-054 (SWCNTs-COOH)</u>: some genes were found to be differentially expressed at all three doses, such as *Serpinb1bc* (8.1, 16.1, 20.9-fold), *Ccl17* (2.5, 5, 6.4-fold), *Retnla* (1.8, 2.2, 2.4-fold), and *Ctsk* (1.6, 2.2, 2.4-fold). Other genes were upregulated and downregulated at medium and high doses. For example, *Slc26a4* (10.1 16.2-fold), *Saa1* (9.2, 14.8-fold), *Orm1* (6.6, 7.5-fold), *Ccl7* (6.1, 7.4-fold), *Cxcl6* (5.5, 8.2-fold), *Fmo3* (-4.3, -3.55-fold), *Pon1* (-2.7, -2-fold), *Gsta5* (-2.4, -

2.5-fold), *Asgr1* (-2.4, -2-fold), and *Galnt15* (-2, -2-fold). Among the large fold changes observed at the highest dose, we found *Serpinb1c* (20.9-fold), *Cxcl6* (8.2-fold), *C15orf48* (7.2-fold), *Ccl17* (6.4-fold), *Ly6a* (5.3-fold), *Fmo3* (-3.5-fold), *Fabp1* (-3-fold), *Gsta5* (-2.5-fold), *Bcan* (-2.4-fold), and *Myh7* (-2.1-fold) (**Table S5d**).

DEGs for MWCNTs

NRCWE-061 (MWCNTs-NH_2): several genes were upregulated in a dose-dependent manner, such as *Slc26a4* (5.8, 8.3, 11.7-fold change), *Serpinb1c* (6.3, 8.1, 9.7-fold change), *Cxcl6* (3.7, 6.5, 8.7-fold change), *Lcn2* (1.8, 3.9, 8.1-fold change), *C15orf48* (2.5, 2.9, 7.7), *Ccl17* (2.7, 4.3, 5.1-fold change), and *Ch25h* (2.2, 3.0, 4.7-fold change). Other DEGs were found at the medium dose, and their fold changes increased at the highest dose. For example, *Saa3* (6.8, 97.8-fold), *Saa1* (2.8, 15.0-fold), *Timp1* (4.2, 15.0-fold), and *Ccl24* (4.6, 13.0-fold). At the medium dose, *Nr1d1* (-1.5-fold), *Hes2* (-1.5-fold), *Nfatc2ip* (-1.5-fold), *Slc16a11* (-1.7-fold) and *Dbp* (-2.8-fold) were upregulated. Among the downregulated genes at the highest dose, we found *Gsta5* (-2.9-fold), *Fabp1* (-3.4-fold), *Scgb3a2* (-3.8-fold), *Pon1* (-3.8-fold), and *Fmo3* (-4.9-fold) (**Table S6a**).

NRCWE-062 (MWCNTs-pristine): some DEGs found at the medium dose showed higher fold change values at the high dose. Among those DEGs were *Saa3* (13.6, 120-fold), *Saa1* (3.4, 20.3-fold), *Timp1* (4.8, 15.7-fold change), *Cxcl6* (6.8, 13.7-fold), *Ccl24* (3.6, 13.3-fold), *Slc26a4* (6.3, 11.1-fold) and *Serpinb1c* (6.5, 10.6-fold). At the medium dose, significant differential expression were observed for downregulated DEGs, such as *Arntl* (-1.5-fold), *Cst8* (-1.6-fold), *Rnase2* (-1.6-fold), *Myh7* (-1.6-fold), and *Ear2* (-1.8-fold). For the highest dose, *Fabp1* (-3.7-fold), *Cidec* (-3.0-

fold), *Fmo3* (-2.9-fold), *Cox8b* (-2.5-fold), and *Fabp3* (-2.4-fold) showed the largest fold changes for downregulated DEGs (**Table S6b**).

NRCWE-063 (MWCNTs-OH): some of the larger fold changes found at the low dose belonged to *Dbp* (4.2-fold), *Per2* (3.0-fold), *Npr3* (2.4-fold), *Tspan4* (2.3-fold), *Per3* (2.3-fold), *Acot1* (-2.0-fold), *Gdpd2* (-2.2-fold), *Arntl* (-2.7-fold), *Angptl4* (-2.7-fold), and *Spon2* (-3.2-fold). At the medium dose, *Dbp* (3.0-fold), *Npr3* (2.7-fold), *Per2* (2.3-fold), *Map3k6* (2.2-fold), *Zbtb16* (2.1-fold), *Klf10* (-1.7-fold), *Rxrg* (-1.7-fold), *Spong2* (-2.0-fold), *Cirbp* (-2.0-fold), and *Arntl* (-2.0-fold) presented significant fold change values. For the highest dose, we also found *Saa3* (29.6-fold), *Cxcl6* (12.4-fold), *Serpinb1c* (9.9-fold), *Slc26a4* (9.0-fold), *Lcn2* (7.4-fold), *Cirbp* (-1.9-fold), *Cst8* (-2.1-fold), *Pon1* (-2.2-fold), *Clstn2* (-2.3-fold) and *Fmo3* (-2.5-fold) to be differentially expressed (**Table S6c**).

NRCWE-064 (MWCNTs-COOH): Some DEGs were found at the medium dose and their fold change increased at the highest dose, such as *Cxcl6* (4.5, 14.6-fold), *Lcn2* (2.2, 8.9-fold), *Ccl17* (5.1-fold), and *Ch25h* (3.5-fold). Some of the larger fold changes found at the low dose were *Dbp* (3.1-fold), *Nr1d1* (1.9-fold), *Hlf* (1.9-fold), *Per3* (1.8-fold), *Tef* (1.7-fold), *Pde4b* (-1.9-fold), *Rasl11a* (-2.0-fold), *Arntl* (-2.0-fold), *Spon2* (-2.1-fold), and *Angptl4* (-2.7-fold). At medium dose *Egr1* (2.3-fold), *Carmn* (1.8-fold), *Il4i1* (1.8-fold), *Kcnj2* (-1.7-fold), *Sfpq* (-1.7-fold), *Col3a1* (-1.7-fold), *Ai314278* (-2.0-fold), and *Acss2os* (-2.3-fold) were also found. At the highest dose, some of the DEGs with the highest fold changes were *Saa3* (61.2-fold), *Slc26a4* (10.1-fold), *Timp1* (9.7-fold), *Ccl24* (9.2-fold), *Serpinb1c* (9.2-fold), *Ear2* (-2.0-fold), *Acot1* (-2.1-fold), *Fmo3* (-2.2-fold), *Cidec* (-2.7-fold), and *Fabp1* (-2.8-fold) (**Table S6d**).

Gene ontology analysis of biological processes

DAVID bioinformatics tool was employed to identify the biological processes enriched by exposure to CNTs. The first analysis was conducted only on upregulated DEGs and the processes with more than five genes (fold change ≥ 1.5) and an FDR *p*-value ≤ 0.05 were included. Another analysis was conducted with DEGs showing fold changes < −1.5, but no significant biological processes were found.

SWCNTs

Biological processes related to the inflammatory response (GO: 0006954), immune response (GO: 0006955), chemotaxis, and chemokine-mediated signaling pathway (GO: 0070098) were over-represented for NRCWE-055 (SWCNTs-pristine), NRCWE-056 (SWCNTs-OH), and NRCWE-057 (SWCNTs-COOH) at the highest dose. In addition to inflammatory response, NRCWE-056 (SWCNTs-OH) was associated with DNA replication (GO: 0006260), cell cycle (GO: 0007049), and DNA unwinding involved in DNA replication (GO: 0006268) (**Figure S5**).

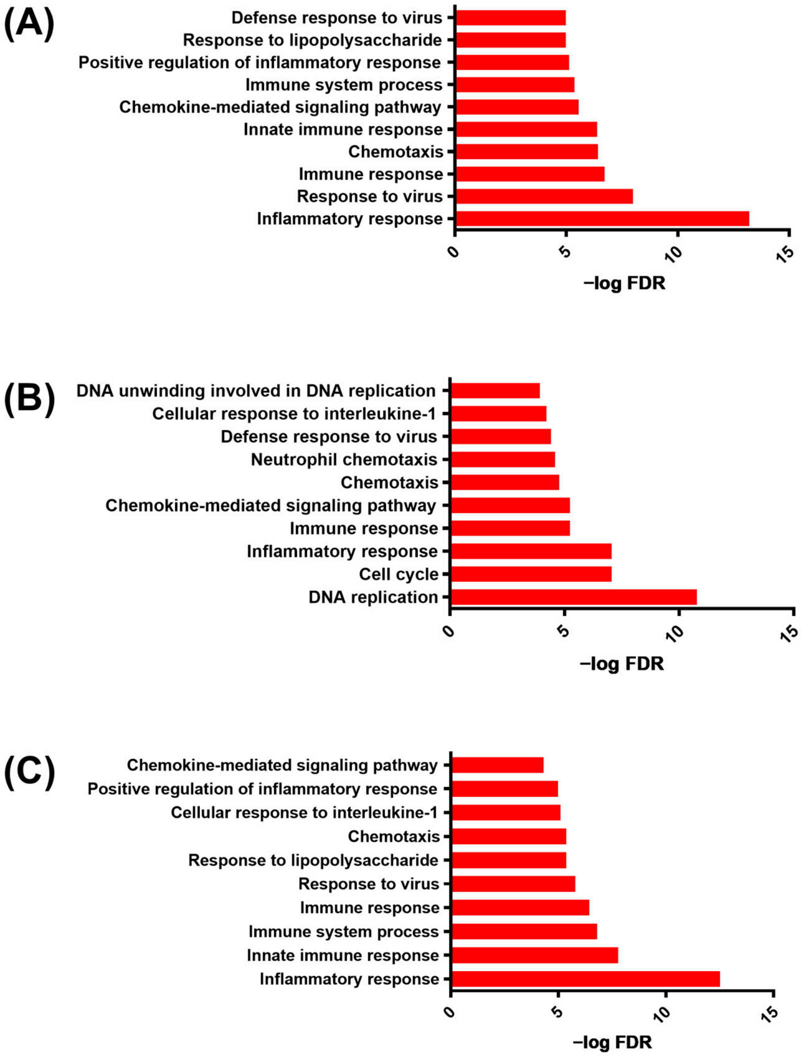

Figure S5. GO enrichment analysis for NRCWE-055, NRCWE-056, and NRCWE-057 by DAVID bioinformatics tool. Top 10 most significant biological processes regulated after 1 day of exposure to (**A**) NRCWE-055 (SWCNTs-pristine), (**B**) NRCWE-056 (SWCNTs-OH), and (**C**) NRCWE-057 (SWCNTs-COOH) at 54 µg/mouse using DAVID bioinformatics based on DEGs FDR $p \leq 0.05$ and fold change ≥ 1.5.

The exposure to NRCWE-051 (SWCNTs-pristine) disturbed several biological processes at the three doses, such as inflammatory response (GO: 0006954), immune response (GO: 0006955), chemotaxis (GO: 0006935), positive regulation of inflammatory response (GO: 0050729), neutrophil chemotaxis (GO: 0030593), and cellular response to interleukin-1 (GO: 0071347). NRCWE-052 (SWCNTs-pristine) did not show any perturbed biological process at the low dose; at the medium dose, it was associated only with inflammatory response (GO: 0006954). At the highest dose, biological processes related to inflammation and DNA replication (GO: 0006260) were over-represented. After exposure to NRCWE-053 (SWCNTs-OH), processes associated with inflammation, DNA replication (GO: 0006260), cell cycle (GO: 0007049), and DNA unwinding involved in DNA replication (GO: 0006268) were disturbed at the high dose. For NRCWE-54 (SWCNTs-COOH), at the medium dose, processes such as inflammatory response (GO: 0050729), cellular response to interleukin-1 (GO: 0071347), cellular response to lipopolysaccharide (GO: 0071222), and monocyte chemotaxis (GO: 0002548) were enriched. The processes of DNA replication (GO: 0006260), immune response (GO: 0006955), chemotaxis (GO: 0006935), inflammatory response (GO: 0006954), chemokine-mediated signaling pathway (GO: 0070098), and neutrophil chemotaxis (GO: 0030593) were perturbed at the medium and high dose. At the high dose, immune system process (GO: 0002376), DNA unwinding involved in DNA replication (GO: 0006268), DNA replication initiation (GO: 0006270), innate immune response (GO: 0045087), and cellular response to interleukin-1 (GO: 0071347) were perturbed (**Figure S6)**.

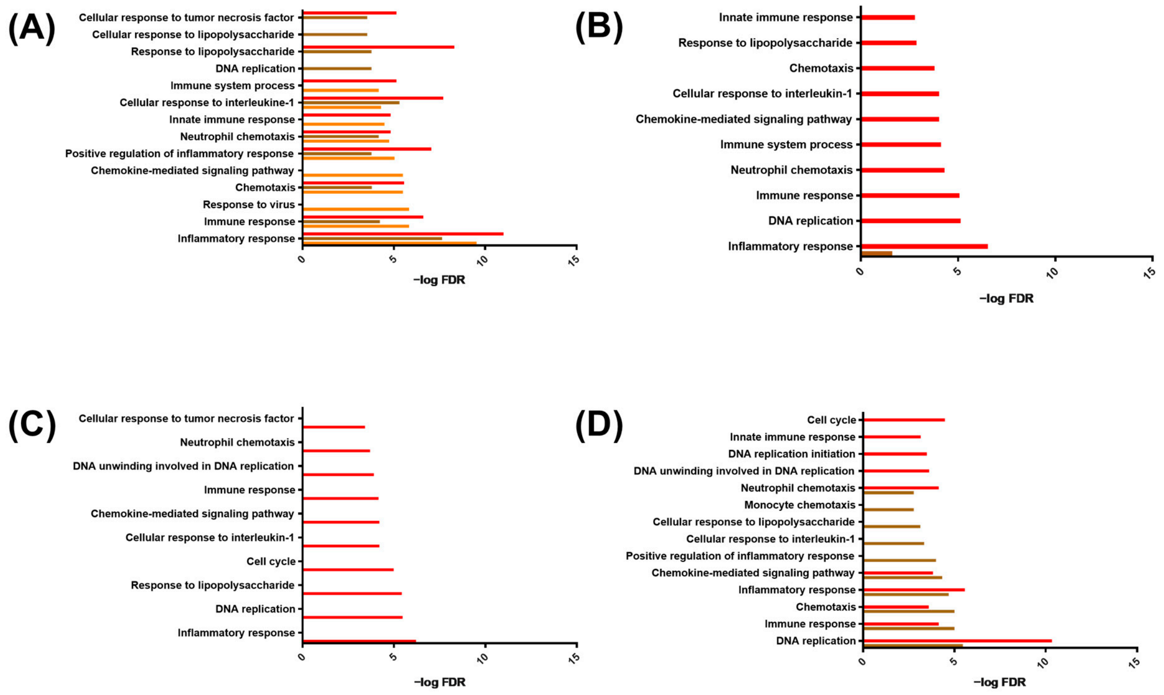

Figure S6. GO enrichment analysis for NRCWE-051, NRCWE-052, NRCWE-053, and NRCWE-054 by DAVID bioinformatics tool. Top 10 most significant biological processes regulated after 1 day of exposure to (**A**) NRCWE-051 (SWCNTs-pristine), (**B**) NRCWE-052 (SWCNTs-pristine), (**C**) NRCWE-053 (SWCNTs-OH), and (**D**) NRCWE-054 (SWCNTs-COOH) using DAVID bioinformatics based on DEGs FDR $p \leq 0.05$ and fold change ≥ 1.5. Orange bars: 6 μg/mouse, brown bars: 18 μg/mouse, red bars: 54 μg/mouse.

MWCNTs

For NRCWE-061 (MWCNTs-NH_2), some enriched biological processes were shared between the medium and high dose: Inflammatory response (GO: 0006954), neutrophil chemotaxis (GO: 0030593), immune response (GO: 0006955), chemotaxis (GO: 0006935), and chemokine-mediated pathway (GO: 0070098). At the high dose, DNA replication (GO: 0006260) and DNA replication initiation (GO: 0006270) were also observed at the highest dose. The exposure to NRCWE-062 (MWCNTs-pristine) at the medium and high dose shared several biological processes such as chemokine-mediated signaling pathway (GO: 0070098), cellular response to interleukin-1 (GO: 0071347), chemotaxis (GO: 0006935), and inflammatory response (GO: 0006954). DNA replication (GO: 0006260) was also perturbed in the high dose group. Among the top biological processes regulated after exposure to NRCWE-063 (MWCNTs-OH), we found the rhythmic process (GO: 0048511) and circadian rhythm (GO: 0007623) at low and medium doses. At the highest dose, immune response (GO: 0006955), inflammatory response (GO: 0006954), chemotaxis (GO: 0006935), chemokine-mediated signaling pathway (GO: 0070098), and neutrophil chemotaxis (GO: 0030593) were over-represented. For NRCWE-064 (MWCNTs-COOH), rhythmic process (GO: 0048511), transcription, DNA-templated (GO: 0006351), and regulation of transcription, DNA-templated (GO: 0006355) were perturbed at the low dose. No processes were found for the medium dose. Inflammatory response (GO: 0006954), chemokine-mediated signaling pathway (GO: 0070098) and immune response (GO: 006955) are some of the processes perturbed at the highest dose (**Figure S7**).

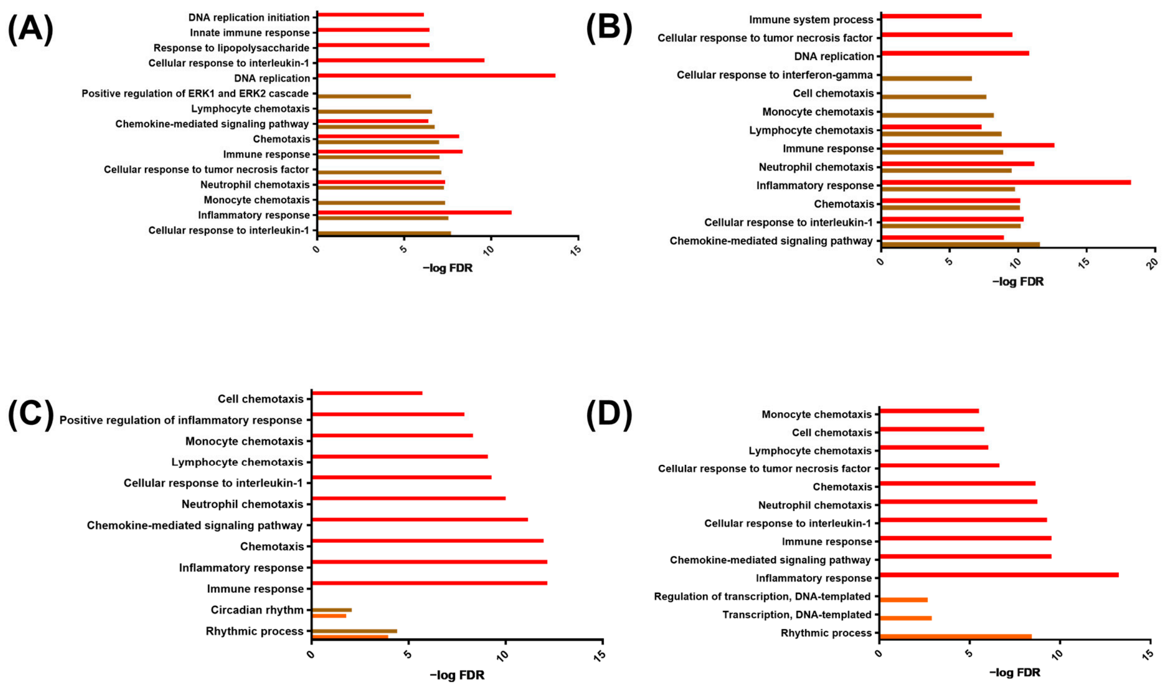

Figure S7. GO enrichment analysis for NRCWE-061, NRCWE-062, NRCWE-063, and NRCWE-064 by DAVID bioinformatics tool. Top 10 most significant biological processes regulated after 1 day of exposure to (**A**) NRCWE-061 (MWCNTs-NH_2), (**B**) NRCWE-062 (MWCNTs-pristine), (**C**) NRCWE-063 (MWCNTs-OH), and (**D**) NRCWE-064 (MWCNTs-COOH) using DAVID bioinformatics based on DEGs FDR $p \leq 0.05$ and fold change ≥ 1.5. Orange bars: 6 µg/mouse, brown bars: 18 µg/mouse, red bars: 54 µg/mouse.

Comparison of DEGs implicated in the common perturbed canonical pathways

At 18 µg/mouse, five canonical pathways (acute phase response signaling, agranulocyte adhesion, and diapedesis, granulocyte adhesion and diapedesis, IL-17 signaling) were shared by NRCWE-051 (SWCNTs-pristine), NRCWE-054 (SWCNTs-COOH), NRCWE-061 (MWCNTs-NH_2), and NRCWE-062 (MWCNTs-pristine). **Figure S8** shows the comparison of upregulated and downregulated DEGs in each pathway for those CNTs. In general, those canonical pathways shared several DEGs among the four CNT types. Acute-phase response signaling was activated through *Saa1*, *C3*, *Serpina3*, *Cfb*, and *Itih4*. For agranulocyte/granulocyte adhesion and diapedesis,

Cxcl6, *Ccl7*, *Ccl17*, *Ccl8*, *Ccl9*, *Cxcl3*, and *Cxcr1* were upregulated. *Cll17* and *Lcn2* were upregulated in IL-17 signaling. *Saa1*, *C3*, *Cd14*, and *Itih4* were upregulated for LXR/RXR activation. NRCWE-051 (SWCNTs-pristine) upregulated and downregulated more DEGs in each canonical pathway.

The comparison of DEGs in each pathway shared for all CNTs at the 54 µg/mouse is shown in **Figure S9**. Several genes are shared among CNT types in those canonical pathways such as *Saa1*, *Il-33*, *Il-6*, *C3*, *Serpina3*, *Cfb*, and *Itih4* for acute phase response signaling; *Cxcl6*, *Ccl7*, *Il-33*, *Ccl2*, *Ccl17*, *Cxcl10*, and *Ccl8* for agranulocyte/granulocyte adhesion and diapedesis; *Saa1*, *Il-33*, *Il-6*, *C3*, *Itih4*, and *Cd14* for LXR/RXR activation; *Il-33*, *Il-6*, *Cd14* (IL-10 signaling), *Il-6*, *Arg1*, *Cxcl12*, and *Mmp3* for tumor microenvironment pathway, *Cxcl6*, *Cxcl10* and *ll-16* for the role of IL-17 in allergic inflammatory airway diseases.

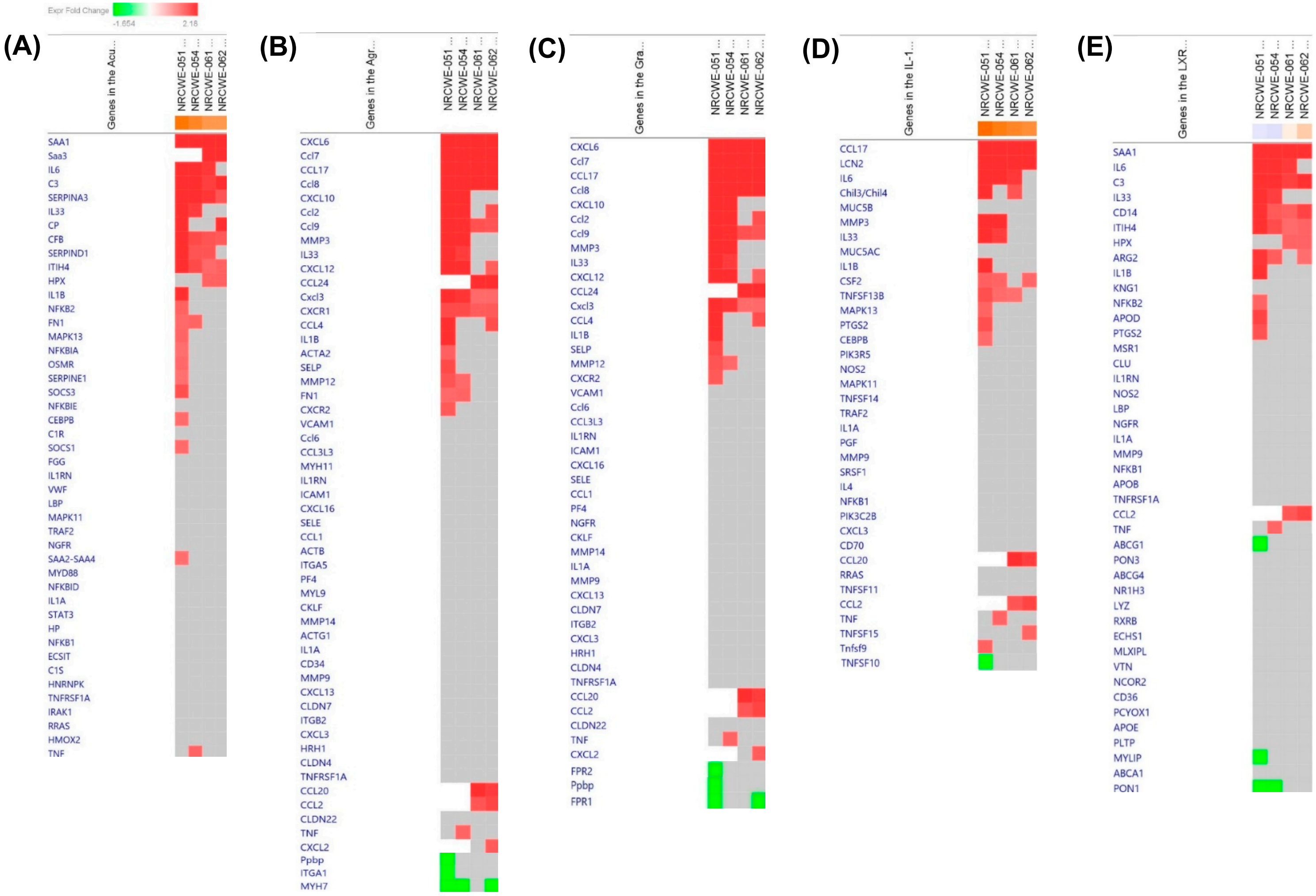

Figure S8. Comparison analysis of DEGs implicated in regulated canonical pathways after 1 day of exposure to CNTs at 18 µg/mouse. (**A**) Acute phase response signaling, (**B**) Agranulocyte adhesion and diapedesis, (**C**) Granulocyte adhesion and diapedesis, (**D**) IL-17 signaling, (**E**) LXR/RXR activation. IPA analysis. Red: upregulated, green: downregulated, grey: in the dataset but did not pass cutoffs and filters, white: not in the dataset. Orange or blue squares indicate predicted activation or predicted inhibition, respectively.

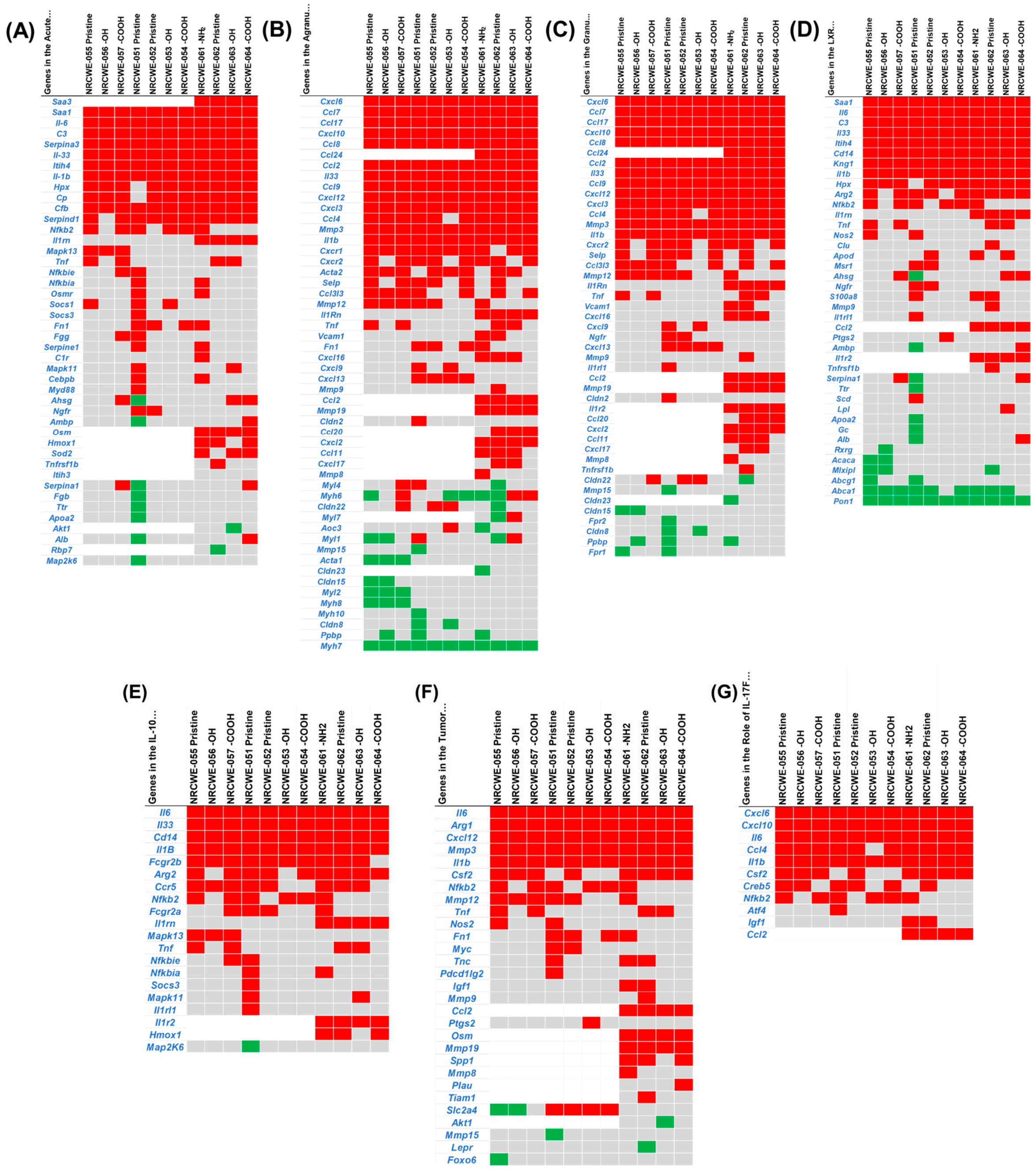

Figure S9. Comparison analysis of DEGs implicated in regulated canonical pathways after 1 day of exposure to CNTs at 54 µg/mouse. (**A**) Acute phase response signaling, (**B**) Agranulocyte adhesion and diapedesis, (**C**) Granulocyte adhesion and diapedesis, (**D**) LXR/RXR activation, (**E**) IL-10 signaling, (**F**) Tumor microenvironment pathway, (**G**) Role of IL-17F in allergic inflammatory airway diseases. IPA analysis. Red: upregulated, green: downregulated, grey: in the dataset but did not pass cutoffs and filters, white: not in the dataset.

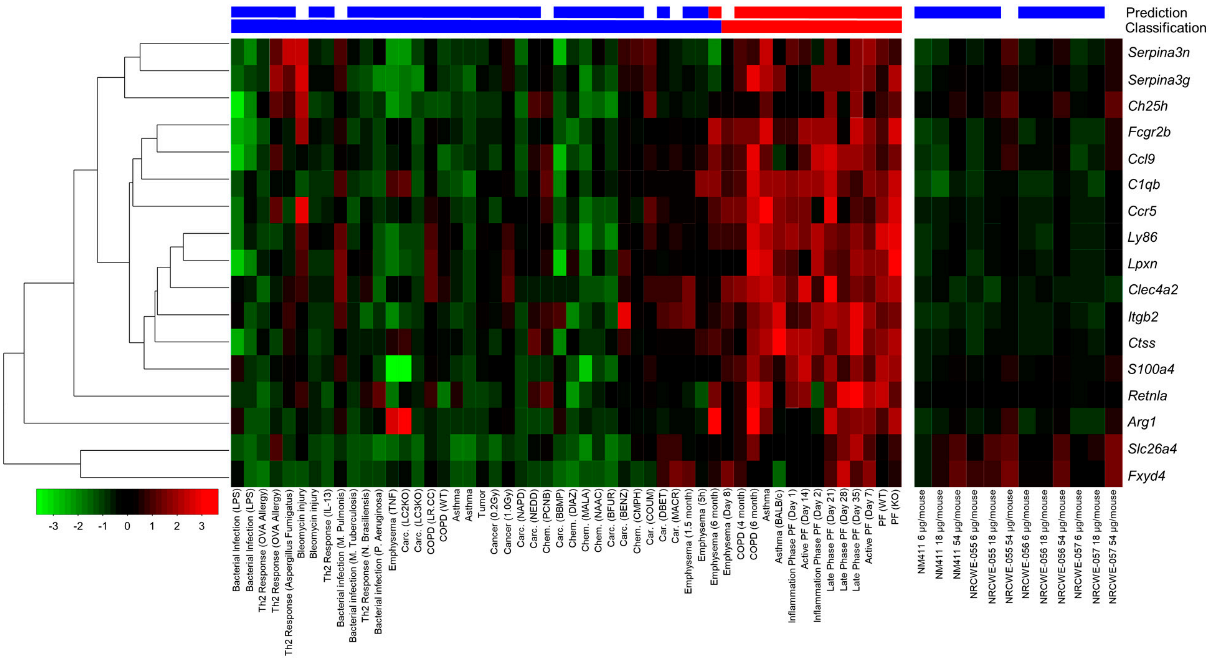

Figure S10. Pro-fibrotic transcriptomic signature after 1 day of exposure to NM-411, NRCWE-055, NRCWE-056, and NRCWE-057. A heatmap that compares the transcriptomic profiles of lung-specific diseases vs. pro-fibrotic transcriptomic signature (PFS17) [14] of lungs exposed to NM-411, NRCWE-055, NRCWE-056, and NRCWE-057. Classification and prediction results are presented with blue (no-fibrotic), red (pro-fibrotic), and no color (unknown). The bottom bar represents the pre-classification of each study based on the literature data, and the top bar represents outcomes of prediction using PFS17. As described in Williams and Halappanavar et al. [67], biclusters related to pulmonary diseases were identified from public microarray datasets. The pro-fibrotic signature was derived from one of the identified bi-clusters. Red: upregulated. Green: downregulated. Table S3 shows the list of pulmonary diseases identified from public microarray datasets [14].

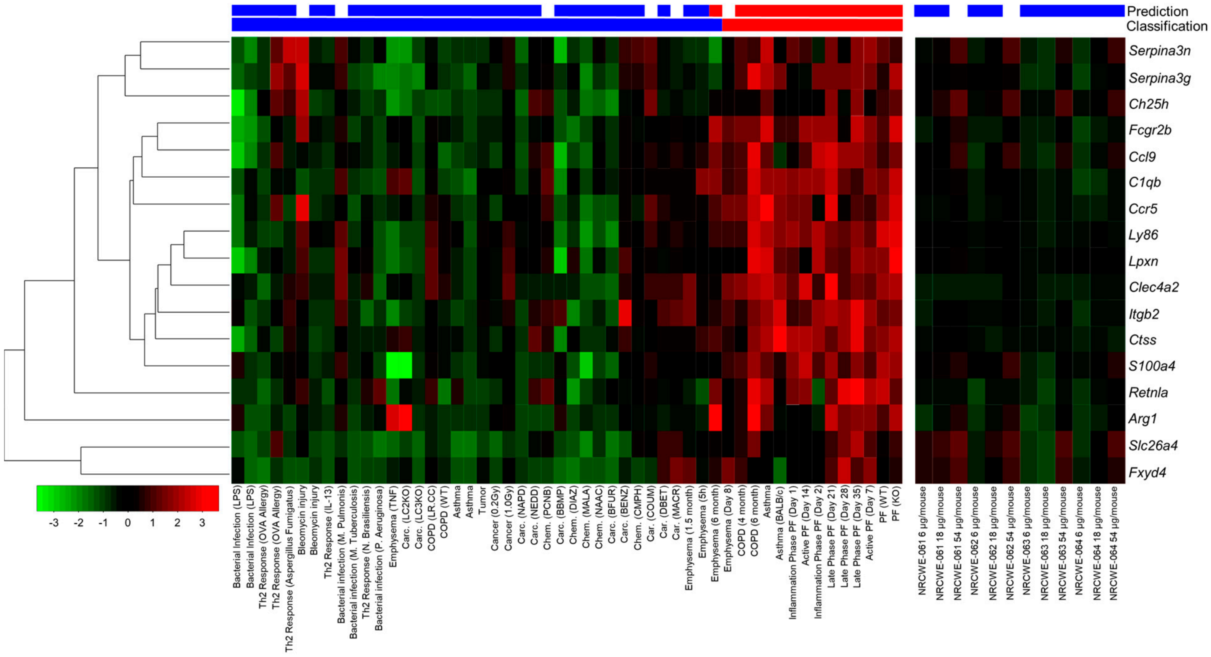

Figure S11. Pro-fibrotic transcriptomic signature after 1 day of exposure to NRCWE-061, NRCWE-062, NRCWE-063, and NRCWE-064. A heatmap that compares the transcriptomic profiles of lung-specific diseases vs. pro-fibrotic transcriptomic signature (PFS17) [14] of lungs exposed to NRCWE-061, NRCWE-062, NRCWE-063, and NRCWE-064. Classification and prediction results are presented with blue (no-fibrotic), red (pro-fibrotic), and no color (unknown). The bottom bar represents the pre-classification of each study based on the literature data, and the top bar represents outcomes of prediction using PFS17. As described in Williams and Halappanavar et al. [67], biclusters related to pulmonary diseases were identified from public microarray datasets. The pro-fibrotic signature was derived from one of the identified bi-clusters. Red: upregulated. Green: downregulated. Table S3 shows the list of pulmonary diseases identified from public microarray datasets [14].

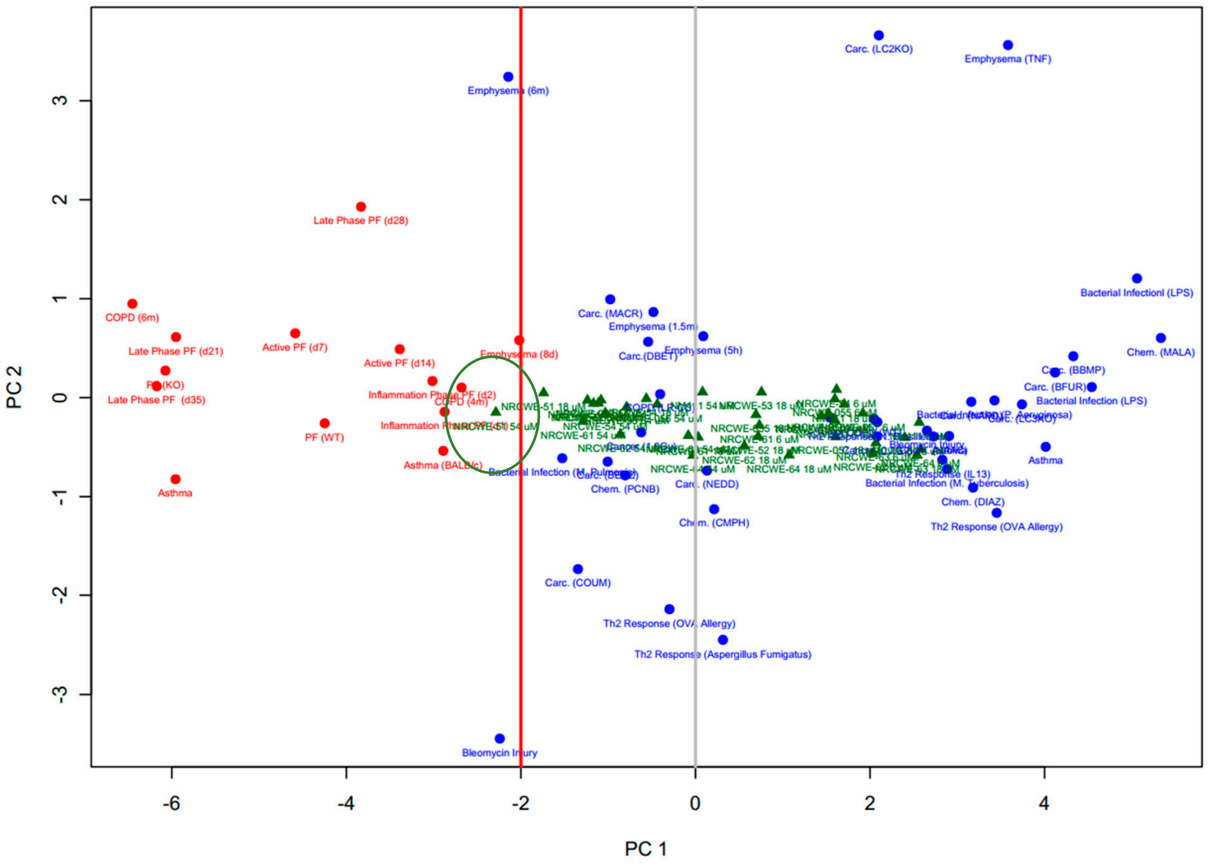

Figure S12. Scatter plot representing the two components of PCA performed using the public microarray datasets described in Table S3. The PCA was performed using the prcomp function in R using the dataset of publicly available *in vivo* lung transcriptomic studies described in Rahman et al. [14] and the dataset produced in this study. The grey line separates profiles that are borderline pro-fibrotic with their first principal component being negative. The red line shows the contrast between the pro-fibrotic and no-fibrotic classes. Red circles: pro-fibrotic data set, blue circles: no pro-fibrotic data set, green triangles: 12 CNTs data set. Green circle: NRCWE-051 at 54 µg/mouse. PC: Principal component. 1 Day post-exposure.
